# The Clinico-Epidemiological Profile and Effectiveness of Codeine and Triprolidine in Dry Cough Patients Not Responding to Non-Opioid-Based Antitussives: Results From a Prospective, Multicenter, Single-Arm, Open-Label Postmarketing Observational Study

**DOI:** 10.7759/cureus.101883

**Published:** 2026-01-20

**Authors:** Deepak Talwar, Balamurugan Santhalingam, Rajveer Kuldeep, Manish K Jain, Gajendra Vikram Singh, Sushant Muley, Ankit Kumar, Urvi K Mistry, Swapnil Deshpande, Shivani Acharya, Rhutuja Rane

**Affiliations:** 1 Medicine, Metro Hospital, Noida, IND; 2 Respiratory Medicine, Chest and Diabetes Research Institute, Chennai, IND; 3 Pulmonology, Jawaharlal Nehru Medical College, Ajmer, IND; 4 Pulmonology, Maharaja Agrasen Super Specialty Hospital, Jaipur, IND; 5 Tuberculosis &amp; Respiratory Diseases, Sarojini Naidu Medical College, Agra, IND; 6 Pulmonary Medicine, Dr Dande Hospitals, Nagpur, IND; 7 Respiratory Medicine, King George's Medical University, Lucknow, IND; 8 Medicine, Abbott Healthcare Pvt. Ltd, Mumbai, IND; 9 Medical Affairs, Abbott Healthcare Pvt. Ltd, Mumbai, IND; 10 Clinical Development and Operations, Abbott Healthcare Pvt. Ltd, Mumbai, IND

**Keywords:** antitussives, codeine phosphate, dry cough, fixed dose combination, triprolidine hcl

## Abstract

Background: Dry cough is highly prevalent in clinical practice, and antitussives remain the cornerstone of treatment. Among these, the combination of codeine phosphate and first-generation antihistamine triprolidine hydrochloride (triprolidine HCl) is often preferred in a dry cough unresponsive to non-opioid antitussives. However, there is limited real-world evidence regarding the effectiveness and safety of the fixed-dose combination (FDC) of codeine phosphate and triprolidine HCl in the Indian population.

Methods: This prospective, multicenter, single-arm, open-label, post-marketing observational study evaluated the clinico-epidemiological profile of Indian patients with dry cough unresponsive to non-opioid antitussives, prescribed FDC codeine phosphate 10 mg + triprolidine HCl 1.25 mg per 5 mL oral syrup, 10 mL thrice daily for seven days. The study also assessed effectiveness, quality of life (QoL), safety, and tolerability of the FDC.

Results: A total of 250 patients (male:female, 129:121; mean±SD age, 40.8 ± 14.0 years) were observed. Most were graduates (107 (42.8%)), with homemakers comprising the largest occupational group (98 (39.2%)). Acute cough was observed in 137 (54.8%) of patients, with upper respiratory tract infection (URTI) as the leading etiology in 188 (75.2%) of patients. From baseline to end of study, mean±SD cough severity decreased by 3.8±1.8, frequency by 2.4±1.2, and nighttime awakening by 3.5±1.8 points (p<0.001), while QoL scores improved by 33.5±19.9 points (p<0.001). Additionally, subgroup analyses also showed significant improvements across etiologies. Treatment was well tolerated, with 15 patients reporting 17 adverse drug reactions (ADRs; mostly constipation and sedation). All ADRs were mild and non-serious in nature and did not lead to treatment discontinuation.

Conclusion: In this observational study of 250 adults with dry cough, balanced by gender and predominantly middle-aged with a mean age 40.8 years, the FDC of codeine phosphate 10 mg and triprolidine HCl 1.25 mg per 5 mL oral syrup, prescribed as 10 mL thrice daily for seven days, significantly reduced cough severity, frequency, and nighttime awakenings, improving the QoL. Importantly, these results support the role of this FDC in the management of dry cough, especially when non-opioid antitussives are ineffective, a key differentiator of this study. Benefits were consistent across different etiologies of dry cough, with only mild, non-serious adverse events.

## Introduction

Cough is often observed as a predominant indicator of respiratory diseases and is among the most common clinical presentations encountered in primary care settings and outpatient departments worldwide [1]. It serves as a vital reflex mechanism that expels irritants from the respiratory tract and may present as either a wet cough (with sputum) or a dry cough (without sputum) [1]. A wet cough is typically associated with infections, inflammation, or chronic conditions such as chronic obstructive pulmonary disease (COPD), whereas a dry cough may arise from asthma or other respiratory disorders [2-4]. Globally, approximately 9.65% of adults are affected by cough, with a higher prevalence reported in Europe, the Americas, and Australia (10-20%), followed by Asia (<5%) [5]. In India, cough is a frequent complaint, reported in nearly 30% of patients visiting primary care facilities and accounting for 6.5% of outpatient consultations, with an estimated overall prevalence of 5% [5-7].

Differentiating the etiology of cough is crucial for accurate diagnosis, monitoring disease progression, and designing appropriate therapeutic strategies [8]. For example, timely identification of specific cough characteristics, such as a wet cough in COPD or a dry cough in asthma, facilitates appropriate treatment selection, leading to improved outcomes and potential prevention of hospitalization [9,10]. Clinical guidelines broadly classify cough based on its duration as acute (<3 weeks), subacute (3-8 weeks), or chronic (>8 weeks), and also specify recommended diagnostic approaches for each category [11,12]. 

Acute cough is often associated with viral upper respiratory tract infections (URTIs); however, sub-acute and chronic coughs may have varied causative factors [11,12]. Subacute cough is commonly caused by factors like chronic rhinosinusitis and active asthma [13], and chronic cough may be caused by upper airway cough syndrome (UACS), gastroesophageal reflux disease (GERD), or cough-variant asthma [14]. UACS-mediated cough is caused by rhinosinusitis, and intranasal corticosteroids, oral antihistamines, or nasal ipratropium bromide are recommended for its management [15,16]. Chronic cough due to GERD is reported in 40% of the patients, and prokinetic agents like metoclopramide, and an acid suppressant with a proton pump inhibitor, along with an anti-reflux diet and lifestyle changes are recommended for its management [17,18]. Cough-variant asthma often occurs at night, and suitable treatment options include bronchodilators, antileukotrienes, theophylline, or oral corticosteroids [18,19]. The usual therapeutic approach for dry cough management recommends antitussive therapies during unexplained cough reflex [20]. Antitussives may act centrally or peripherally [21]. Centrally acting antitussives like codeine, dextromethorphan, and morphine suppress the cough reflex by inhibiting the cough center in the brain. This action reduces the nerve impulses sent to the muscles responsible for producing a cough [21]. Codeine is a commonly used centrally acting cough suppressant known for its efficacy in controlling both disease-mediated and unexplained chronic cough, with the added benefit of analgesia and sedation [22]. Most cough formulations are combinations of antitussives with other agents like antihistamines for enhanced symptomatic relief [21]. Triprolidine, a first-generation antihistamine, has been proven effective in combination therapy for symptomatic relief of coughs and colds [23]. In fact, fixed-dose combination (FDC) therapy is considered a rational approach in cough management, as it enhances treatment efficacy through complementary mechanisms while minimizing pill burden and improving patient adherence [7]. Such combinations, especially those involving a centrally acting antitussive such as codeine and a first-generation antihistamine such as triprolidine, have demonstrated both clinical effectiveness and tolerability in managing dry cough [22,24].

Despite the long-standing clinical use of the combination of codeine phosphate and triprolidine hydrochloride (HCl), there is limited real-world evidence on their use in the Indian population. The widespread use of codeine in the country highlights an urgent need to better characterize patient profiles and evaluate treatment outcomes in this demographic. Limited clinical data on the effectiveness and safety of FDCs in Indian patients underscores the need for studies to inform clinical decision-making and optimize treatment strategies. Therefore, the present study aimed to evaluate the effectiveness and safety of the FDC of codeine phosphate 10 mg and triprolidine HCl 1.25 mg per 5ml syrup, prescribed at a dose of 10mL thrice daily for a period of seven days, in the management of Indian patients with dry cough not responding to non-opioid based antitussives.

## Materials and methods

Study design

This prospective, multicenter, single-arm, open-label, post-marketing observational study (CTRI/2024/12/078427; date of registration 23 December 2024) evaluated the demographic and clinico-epidemiological profile of Indian patients prescribed an FDC of codeine phosphate 10 mg and triprolidine HCl 1.25 mg per 5 mL syrup, at a dose of 10 mL thrice daily for a period of seven days, and assessed the effectiveness and safety of the FDC in the management of patients with dry cough, not responding to non-opioid-based antitussive. The study was conducted from December 2024 to June 2025 at seven sites across different geographical locations in India.

The study was conducted in accordance with ICH-GCP guidelines, the Declaration of Helsinki, applicable local regulations, including the New Drugs and Clinical Trials Rules, 2019, and institutional SOPs. The protocol, informed consent form, and amendments were approved by the relevant ethics committee before study initiation.

Patients

The inclusion criteria comprised patients aged ≥18 years, with a clinical diagnosis of dry cough for at least one week, not responding to non-opioid-based antitussives, and were prescribed the study FDC of codeine phosphate and triprolidine HCl for the symptomatic relief of dry cough associated with any etiology, by the treating physician as per the approved label. All patients were required to provide written informed consent and comply with study procedures.

Exclusion criteria included patients with contraindications to codeine phosphate or triprolidine HCl, or a history of hypersensitivity or idiosyncratic reaction to any of the product ingredients. Patients with pre-existing conditions such as respiratory depression, COPD, or an asthma attack were excluded. Those with a history of alcohol intoxication, narrow-angle glaucoma, urinary retention, raised intracranial tension, head injury, peptic ulcer, paralytic ileus, intestinal obstruction, biliary or gallbladder disorders, severe hypertension, or severe coronary artery disease were also ineligible. In addition, patients with severe hepatic or renal impairment, those receiving monoamine oxidase inhibitor (MAOI) therapy or within two weeks of discontinuing MAOIs, and known CYP2D6 ultra-rapid metabolizers were excluded. Women who were pregnant, planning pregnancy, unwilling to use contraception, or lactating, and newborns or premature infants were also not eligible.

Study endpoints

This study aimed to evaluate the demographic and clinico- epidemiological profile of Indian patients prescribed a FDC of codeine phosphate 10 mg and triprolidine HCl 1.25 mg per 5 mL syrup, at a dose of 10 mL thrice daily for a period of seven days, and to assess the effectiveness and safety of this FDC in managing dry cough in patients unresponsive to non-opioid antitussives.

The primary endpoint of the study was the assessment of clinico-epidemiological characteristics of the enrolled population (safety set), including demographics (age, sex, education, occupation) and clinical presentation such as cough etiology, cough classification, cough severity measured by visual analog scale (VAS), cough frequency measured by 7-point Likert scale, and nighttime awakening measured by VAS.

The secondary endpoints (effectiveness) were the changes from baseline to Day 7 (±2 days) in cough severity using VAS, cough frequency using 7-point Likert scale, nighttime awakening using VAS, and patient quality of life (QoL) using the Leicester Cough Questionnaire (LCQ)-acute score, following thrice-daily treatment with the study FDC for seven days [25]. Formal permission to use the LCQ-Acute in this study was obtained from the copyright holder, Birring Medical Services Ltd., Dartford, Kent, United Kingdom, following payment of the required licensing fee.

The exploratory endpoints assessed the same parameters grouped by cough etiology, evaluating mean changes from baseline to Day 7 (±2 days) post-treatment. Safety outcomes encompassed the occurrence of adverse drug reactions (ADRs), other pharmacovigilance-relevant information (OPRI), serious ADRs and OPRI, as well as ADRs resulting in treatment discontinuation during the seven days of treatment with the study FDC of codeine phosphate 10 mg and triprolidine HCl 1.25 mg per 5 mL syrup.

Statistical analysis

The primary objective of the study was to describe the clinico-epidemiological profile of patients with dry cough; no formal sample size calculation was performed for this purpose. For the secondary objective of evaluating efficacy, the study aimed to detect a significant increase from baseline in total LCQ score after seven days. Based on literature, SD of the change from baseline in total LCQ score was estimated at 12 [26]. With 210 evaluable patients, the study had 95% power to detect a 3-point increase at a 5% significance level. To account for potential dropouts and ensure sufficient evaluable patients for effectiveness outcomes, 250 patients were recruited.

All patients who received at least one dose of the study medication were included in the safety population. Patients in the safety population who had some post-baseline efficacy data were included in the intention-to-treat (ITT) population. The per-protocol (PP) population consisted of patients in the ITT population who completed the study without major protocol deviations and was used for the effectiveness analysis.

Continuous variables for the primary, secondary, and exploratory endpoints were summarized using mean and SD. Categorical variables for the primary endpoint were presented as counts and percentages (n (%)). Safety parameters were summarized using descriptive statistics (frequency (n) and percentage (%)). Changes in continuous outcomes (secondary and exploratory endpoints) from baseline to follow-up were analyzed using paired t-tests with a two-sided significance level of 5%. Mean, SD, and mean change were rounded to one decimal place. All statistical analyses were performed using Statistical Package for the Social Sciences (SPSS) version 26.

## Results

Patient disposition

A total of 250 patients were enrolled in the study. All enrolled patients received the FDC of codeine phosphate 10 mg and triprolidine HCl 1.25 mg per 5 mL syrup, at a dose of 10 mL thrice daily for a period of seven days. Two patients were lost to follow-up; hence, the ITT set comprised 248 patients. Additionally, four patients were reported to be non-compliant with study medication (dose (n=1) and dosage (n=3)), and were not considered for secondary effectiveness analysis, yielding a PP analysis set of 244 patients.

Primary endpoint

The study enrolled 250 patients (male: female, 129:121) with a mean±SD age of 40.8±14.0 years. Among them, 107 (42.8%) were college graduates, 60 (24.0%) were high-school passouts, and 54 (21.6%) had completed an intermediate/diploma. The most common occupation was homemaker/other (98 (39.2%)), followed by professionals (39 (15.6%)) and unemployed (29 (11.6%)).

At baseline, the mean±SD cough severity measured by VAS was 6.2±1.5, cough frequency measured by a 7-point Likert scale was 4.9±0.8, and nighttime awakening measured by VAS was 5.4±1.9. URTI was the most common cause of dry cough (75.2%), with 54.8% classified as acute, 43.6% as subacute, and 1.6% as chronic. The demographic and clinico-epidemiological profile of patients is summarized in Table 1.

**Table 1:** Clinico-epidemiological profile and baseline characteristics of patients – Safety population N: Number of patients in Safety Set; n= Number of patients with data available; Percentages are calculated using 'N' as the denominator; VAS: Visual analog scale; URTI: ; GERD: ; UACS: Upper airway cough syndrome

Parameter	Overall (N = 250)
Sex, n (%)	
Male	129 (51.6)
Female	121 (48.4)
Age (years), mean±SD	40.8±14.0
Cough Severity (VAS), mean±SD	6.2±1.5
Cough Frequency (7-point Likert scale), mean±SD	4.9±0.8
Nighttime Awakening (VAS), mean±SD	5.4±1.9
Duration of Cough (Months), mean±SD	2.5±0.7
Etiology of Cough, n (%)	
URTI	188 (75.2)
UACS	29 (11.6)
Cough-Variant Asthma	17 (6.8)
GERD	09 (3.6)
Unexplained Cough	07 (2.8)
Classification of Cough, n (%)	
Acute Cough	137 (54.8)
Subacute Cough	109 (43.6)
Chronic Cough	04 (1.6)
Education, n (%)	
Uneducated	01 (0.4)
Primary School	07 (2.8)
Professional Degree	10 (4.0)
Middle School	11 (4.4)
Intermediate/Diploma	54 (21.6)
High School	60 (24.0)
Graduate	107 (42.8)
Occupation, n (%)	
Legislators/Senior Officers	02 (0.8)
Plant and Machine Operators	04 (1.6)
Craft and Related Trade workers	04 (1.6)
Skilled Agricultural/Fishery Workers	10 (4.0)
Elementary Occupation	10 (4.0)
Clerks	14 (5.6)
Technician/Associate Professional	16 (6.4)
Skilled Workers/Shop and Market Sales Workers	24 (9.6)
Unemployed	29 (11.6)
Professionals	39 (15.6)
Others (Homemaker etc.)	98 (39.2)

Secondary effectiveness outcome

The mean±SD cough severity score measured using the VAS decreased significantly (p<.001) by 3.8±1.8 from a baseline score of 6.3±1.5. The mean±SD cough frequency score measured using a 7-point Likert scale declined significantly (p<.001) by 2.4±1.2 at Visit 2 (Day 7 ± 2 days), compared to a baseline score of 4.9±0.8. Similarly, the mean±SD nighttime awakening score measured by VAS decreased significantly (p<.001) by 3.5±1.8 at Visit 2, compared to a baseline score of 5.4±1.8. Results as summarized in Table 2.

**Table 2:** Mean±SD change in secondary effectiveness points after seven days of treatment compared to baseline (PP Set, N=244) N=Number of patients in PP Set; CI: Confidence interval; PP: Per protocol; VAS: Visual analog scale ^a^ Analyzed using Paired Sample T-Test

Variables	Visit 1 (Day 1) Mean±SD	Visit 2 (Day 7±2 Days) Mean±SD	t-statistic	Mean±SD Difference (95% CI)	P value^a^
Assessment of Cough Severity (VAS)	6.3±1.5	2.5±2.1	-33.46	-3.8±1.8 (-4.0, -3.6)	p<.001
Cough Frequency (7-point Likert Scale)	4.9±0.8	2.5±1.1	-32.94	-2.4±1.2 (-2.6, -2.3)	p<.001
Nighttime Awakening (VAS)	5.4±1.8	1.9±1.7	-30.25	-3.5±1.8 (-3.8, -3.3)	p<.001

**Figure 1:**
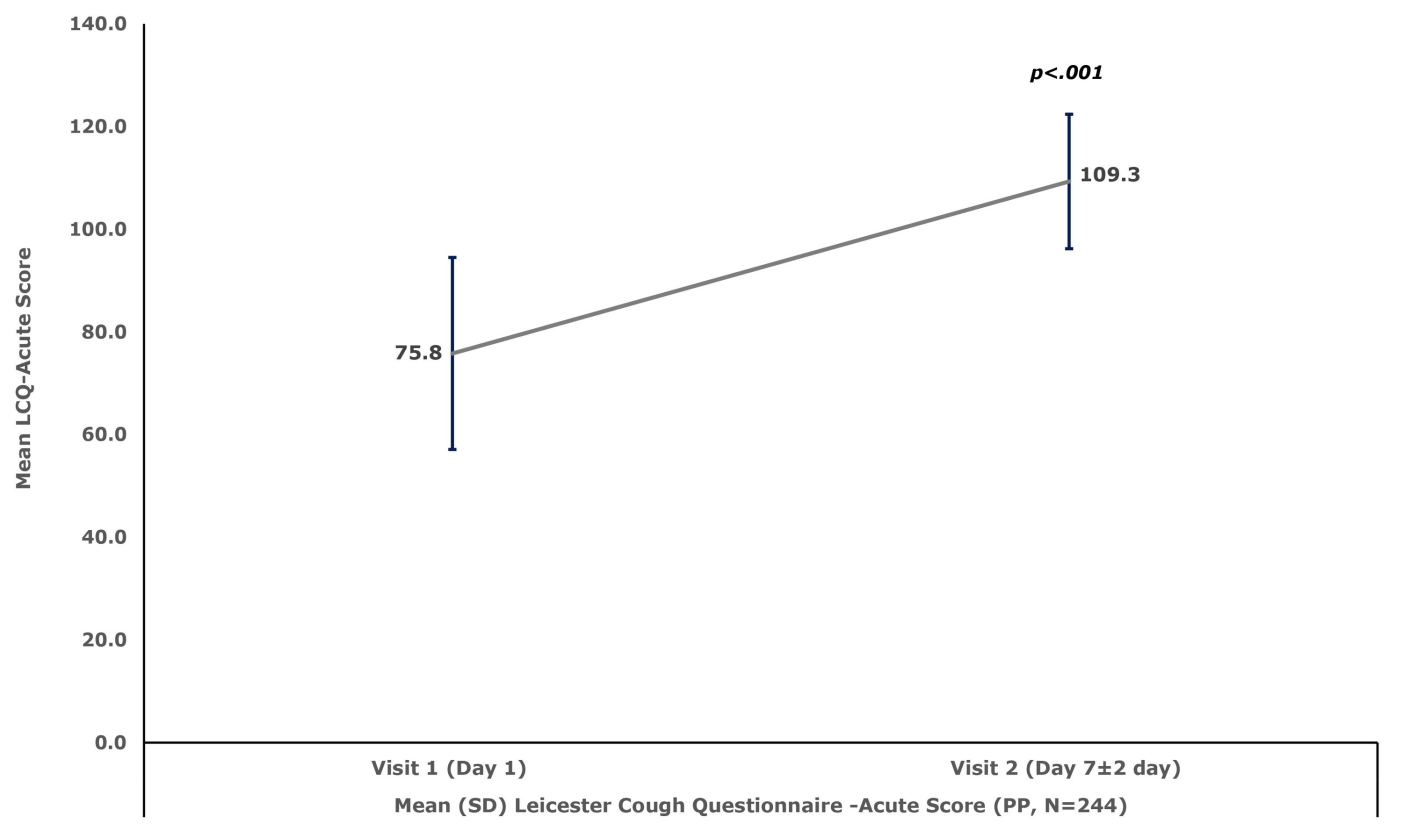
Mean (±SD) QoL measured by LCQ-Acute score – (PP Set, N=244) LCQ-Acute: Leicester Cough Questionnaire-Acute; QoL: Quality of life; PP: Per protocol The use of LCQ-Acute was formally licensed from Birring Medical Services Ltd. *Analyzed using Paired Sample T-Test

This corresponded to a significant improvement in the QoL, as measured by the mean±SD LCQ-Acute score, which increased by 33.5±19.9 (95%CI, 31.0, 36.0; p<.001) to 109.3±13.5 at Visit 2 (Day 7±2 days), from the baseline mean±SD score of 75.8±18.7. The results are illustrated in Figure 1.

Exploratory effectiveness outcome

Compared to the baseline, statistically significant improvements (p-values ranging from <0.05 to <0.001) in the mean (SD) cough severity and nighttime awakening (VAS), cough frequency (7-point Likert scale), and QoL (LCQ-Acute score), were reported in patients of different etiologies of cough. Detailed results, including p-values, are summarized in Table 3.

**Table 3:** Mean±SD change in symptoms by etiology of cough at Visit 2 (Day 7±2) – (PP Set, N=244) N = Number of patients in PP Set; n= Number of patients with data available; GERD: Gastroesophageal reflux disease; LCQ-Acute: Leicester Cough Questionnaire-Acute; PP: Per protocol; URTI: Upper respiratory tract infection; UACS: Upper airway cough syndrome; VAS: Visual analog scale *Analyzed using Paired Sample T-Test

Etiology of Cough (n)	Symptoms	Cough Severity (VAS)	Cough Frequency (7-Point Likert Scale)	Nighttime Awakening (VAS)	LCQ-Acute
URTI (n=183)	Mean±SD Baseline Score	6.3±1.6	4.9±0.9	5.3±2.0	75.9±18.2
	Mean±SD Visit 2 Score	2.3±2.2	2.2±1.0	1.7±1.8	112.7±12.1
	Mean±SD Change from Baseline	-4.0±1.8	-2.7±1.2	-3.6±1.9	36.8±19.6
	P value*	p<.001	p<.001	p<.001	p<.001
UACS (n=28)	Mean±SD Baseline Score	6.1±0.8	5.0±0.9	5.9±1.3	75.9±24.0
	Mean±SD Visit 2 Score	2.6±1.6	3.0±1.1	2.0±1.4	101.1±11.0
	Mean±SD Change from Baseline	-3.5±1.6	-2.0±0.9	-3.9±1.6	25.3±20.2
	P value*	p<.001	p<.001	p<.001	p<.001
Cough-Variant Asthma (n=17)	Mean±SD Baseline Score	6.3±0.8	5.1±0.2	6.3±0.6	79.2±6.4
	Mean±SD Visit 2 Score	3.7±0.8	3.8±0.5	3.4±0.6	100.4±7.0
	Mean±SD Change from Baseline	-2.6±1.2	-1.3±0.6	-2.9±0.9	21.1±7.6
	P value*	p<.001	p<.001	p<.001	p<.001
GERD (n=9)	Mean±SD Baseline Score	6.0±0.9	5.0±0.0	4.3±1.1	84.6±7.6
	Mean±SD Visit 2 Score	3.8±1.0	3.7±0.7	3.1±0.6	99.6±6.9
	Mean±SD Change from Baseline	-2.2±1.4	-1.3±0.7	-1.2±1.4	15.0±6.2
	P value*	p<.01	p<.001	p<.05	p<.001
Unexplained Cough (n=7)	Mean±SD Baseline Score	6.3±0.8	4.9±0.9	6.4±0.8	53.6±25.3
	Mean±SD Visit 2 Score	2.6±1.9	3.0±1.3	2.7±2.0	85.9±11.0
	Mean±SD Change from Baseline	-3.7±1.6	-1.9±0.7	-3.7±1.7	32.3±24.8
	P value*	p<.01	p<.001	p<.01	p<.05

Safety and tolerability

Safety was evaluated through monitoring of all ADRs and or OPRIs. A total of 15 patients (6.0%) experienced 17 ADRs (6.8%). Reported ADRs included constipation and sedation (eight events (3.2%) each) and dizziness (one event (0.4%)). All ADRs were mild and non-serious, and considered potentially related to the study drug. Concomitant medication was prescribed for five ADRs (29.4%), while no action was required for 12 ADRs (70.6%). At the end of the seven-day study, nine ADRs (52.9%) had resolved, and eight (47.1%) were in the process of resolving. No ADRs led to the study drug discontinuation. Overall, 198 (79.8%) patients were fully compliant with the study medication, 46 (18.5%) moderately compliant, and four (1.6%) were non-compliant.

## Discussion

Dry cough is one of the most common symptoms prompting medical consultation globally, and may occur in both healthy and diseased individuals [27]. A dry cough can significantly impair the QoL and often requires targeted symptomatic management with antitussives. Therefore, the current study evaluated the effectiveness and safety of the FDC of codeine phosphate 10 mg and triprolidine HCl 1.25 mg per 5 mL syrup at a dose of 10mL thrice daily for a period of seven days, and demonstrated a significant improvement in cough severity, cough frequency, nighttime awakening score, and overall QoL.

The study enrolled 250 patients, comprising 129 male and 121 female patients, with a mean±SD age of 40.8±14.0 years. Within the study population, 98 patients (39.2%) were homemakers, 39 (15.6%) were professionals, and 29 (11.6%) were unemployed. With respect to educational background, the largest subgroup comprised college graduates (107 (42.8%)), followed by high-school graduates (60 (24.0%)) and individuals with intermediate/diploma qualifications (54 (21.6%)). The most common underlying etiological factor of dry cough was URTI (188 (75.2%)), and the highest proportion of patients suffered from acute dry cough (137 (54.8%)), followed by subacute dry cough (109 (43.6%)) and chronic dry cough (4 (1.6%)).

In the present study, the mean±SD cough severity, measured by VAS score, significantly decreased by 3.8±1.8 points at Visit 2 (Day 7 ± 2 days), as compared to the baseline score of 6.3±1.5. Further, the mean±SD cough frequency score, measured by a 7-point Likert scale, showed a significant drop by 2.4±1.2 points, from the baseline score of 4.9±0.8, at Visit 2 (Day 7 ± 2 days). Similarly, the mean±SD nighttime awakening score, measured by VAS, decreased significantly by 3.5±1.8, compared to a baseline of 5.4±1.8, at Visit 2 (Day 7 ± 2 days). The improvements led to a significant progress in the QoL, as measured by the mean±SD LCQ-Acute score, which significantly increased by 33.5±19.9, from the baseline score of 75.8±18.7 to 109.3±13.5, at Visit 2 (Day 7±2 days). The results of the present study are consistent with those reported by Thomas et al. who observed a significant mean reduction in cough severity, cough frequency, and sleep disruption following a seven-day treatment with 5 mL of codeine phosphate (10 mg) and triprolidine HCl (1.25 mg) syrup prescribed thrice daily in adult patients with dry cough [26]. This suggests that the FDC of codeine phosphate (10 mg) and triprolidine HCl (1.25 mg) may be beneficial not only in patients with dry cough but also in those who do not achieve adequate relief with non-opioid-based antitussives.

Similarly, an investigation into dihydrocodeine monotherapy in a Korean population found a significant decrease in cough severity and improved QoL, particularly among patients with UACS, asthmatic cough, and unexplained cough [28]. Another independent study in lung-cancer patients with dry cough also reported comparable improvements in cough severity and nighttime awakenings after one week of dihydrocodeine monotherapy [29]. Limited data was observed for triprolidine’s direct effect on dry cough; however, insights were drawn from its action on other respiratory conditions. Bye et al. demonstrated that triprolidine monotherapy improved sneezing score in two days in patients with common cold, suggesting its role in managing upper respiratory symptoms [23].

This study demonstrated satisfactory patient compliance with the study FDC prescribed thrice daily for seven days. Overall, 198 (79.8%) of patients were fully compliant, with an additional 46 (18.5%) showing moderate compliance, while only 4 (1.6%) of patients were non-compliant. Safety was assessed by monitoring and documenting all ADRs, OPRIs, serious ADRs/OPRIs, and any events leading to treatment discontinuation. A total of 17 ADRs were observed in 15 patients (6.0%) from the PP set. The most commonly reported ADRs were constipation and sedation, each occurring in 8 (3.2%) of patients. Although these events were considered potentially related to the study drug, they were all mild and non-serious. Concomitant medication was prescribed for five ADRs, while the rest required no intervention. Post-treatment regimen with the FDC, nine ADRs were resolved, and none led to discontinuation of the study. Previous studies on codeine-related ADRs have also reported non-serious events such as drowsiness, headache, constipation, dyspepsia, and somnolence [28-30].

Certain limitations of this study are acknowledged, which include the open-label design, short follow-up duration, and the absence of a control or comparator group, due to which outcome assessment is prone to potential bias. However, using a statistically powered sample size, validated scales, standardized definitions, and the inclusion of relevant clinical characteristics, the study ensures robustness and precision of the results. The study was conducted in routine clinical practice to establish the applicability of the study FDC to real-world scenarios. Such observational studies offer practical insights for informed clinical decision-making. Given the current scarcity of long-term data on effective combination therapies for dry cough, these findings provide important preliminary evidence that can serve as a foundation for the design of future studies.

## Conclusions

In this observational study of 250 adults with dry cough, dry cough was found to be comparable across genders (129 males and 121 females), with a mean age of 40.8 years. Acute cough manifested in more than half of the participants (137 (54.8%)), followed by subacute dry cough (109 (43.6%)) and chronic dry cough (4 (1.6%)). The dry cough was predominantly attributable to URTIs.

The FDC of codeine phosphate 10 mg and triprolidine HCl 1.25 mg per 5 mL oral syrup, prescribed as 10 mL thrice daily for seven days, significantly reduced cough severity, frequency, and nighttime awakenings, improving the QoL. Importantly, these results support the role of this FDC in the management of dry cough, especially when non-opioid antitussives are ineffective, a key differentiator of this study. Benefits were consistent across different etiologies of dry cough. The reported ADRs, mainly constipation and sedation, were mild and non-serious in nature, demonstrating a acceptable safety profile.

Thus, the FDC of codeine phosphate and triprolidine HCl was found to be effective and well-tolerated in the management of dry cough, particularly when non-opioid antitussives are inadequate. The study’s robust methodology provides important preliminary evidence that can serve as a valuable foundation for the design of future long-term studies aimed at addressing the current paucity of data on effective combination therapies for dry cough.
